# Specific Features of the Proteomic Response of Thermophilic Bacterium *Geobacillus icigianus* to Terahertz Irradiation

**DOI:** 10.3390/ijms232315216

**Published:** 2022-12-02

**Authors:** Svetlana Bannikova, Tamara Khlebodarova, Asya Vasilieva, Irina Mescheryakova, Alla Bryanskaya, Elizaveta Shedko, Vasily Popik, Tatiana Goryachkovskaya, Sergey Peltek

**Affiliations:** 1 Federal Research Center Institute of Cytology and Genetics (ICG), Siberian Branch of Russian Academy of Sciences (SB RAS), 10 Lavrentiev Ave., 630090 Novosibirsk, Russia; 2 Kurchatov Genomic Center of Cytology and Genetics (ICG), Siberian Branch of Russian Academy of Sciences (SB RAS), 10 Lavrentiev Ave., 630090 Novosibirsk, Russia; 3 Budker Institute of Nuclear Physics, Siberian Branch Russian Academy of Sciences (SB RAS), 11 Acad. Lavrentieva Pr., 630090 Novosibirsk, Russia

**Keywords:** terahertz radiation, *Geobacillus icigianus*, stress response, non-thermal radiation, proteomics

## Abstract

Studying the effects of terahertz (THz) radiation on the proteome of temperature-sensitive organisms is limited by a number of significant technical difficulties, one of which is maintaining an optimal temperature range to avoid thermal shock as much as possible. In the case of extremophilic species with an increased temperature tolerance, it is easier to isolate the effects of THz radiation directly. We studied the proteomic response to terahertz radiation of the thermophilic *Geobacillus icigianus*, persisting under wide temperature fluctuations with a 60 °C optimum. The experiments were performed with a terahertz free-electron laser (FEL) from the Siberian Center for Synchrotron and Terahertz Radiation, designed and employed by the Institute of Nuclear Physics of the SB of the RAS. A *G. icigianus* culture in LB medium was THz-irradiated for 15 min with 0.23 W/cm^2^ and 130 μm, using a specially designed cuvette. The life cycle of this bacterium proceeds under conditions of wide temperature and osmotic fluctuations, which makes its enzyme systems stress-resistant. The expression of several proteins was shown to change immediately after fifteen minutes of irradiation and after ten minutes of incubation at the end of exposure. The metabolic systems of electron transport, regulation of transcription and translation, cell growth and chemotaxis, synthesis of peptidoglycan, riboflavin, NADH, FAD and pyridoxal phosphate cofactors, Krebs cycle, ATP synthesis, chaperone and protease activity, and DNA repair, including methylated DNA, take part in the fast response to THz radiation. When the response developed after incubation, the systems of the cell’s anti-stress defense, chemotaxis, and, partially, cell growth were restored, but the respiration and energy metabolism, biosynthesis of riboflavin, cofactors, peptidoglycan, and translation system components remained affected and the amino acid metabolism system was involved.

## 1. Introduction

One of the conditions of modern life development on planet Earth is the presence of water in both liquid (e.g., world oceans, rivers, and lakes) and gas phases. Since terahertz (THz) irradiation is almost fully absorbed by the atmosphere and water, living organisms in the evolution process did not develop protection mechanisms from it. The frequency spectrum of THz irradiation falls between the infra-red and high-frequency ranges, with a wavelength from 0.01 to 3 mm. Due to the appearance of new emitters and detection systems, humanity began to utilize the THz electromagnetic frequency range in scientific and applied operation fields, which suggests that more and more humans are interacting with it, especially in security systems and when using diagnostic medical appliances.

In this respect, there is an urgency to conduct research on the impact of terahertz irradiation on the metabolic nodes of living organisms. THz irradiation is suitable for the role of a model impact factor on living systems—it is possible to conduct long and pulsed THz irradiation in a wide range of wavelengths, doses, and irradiation conditions. The low quant energy (from 0.4 to 40 milli-electron volts) of THz irradiation cannot lead to atom ionization, and thus cause well-known circumstances of living organisms that are in contact with a different, higher energy range of electromagnetic radiation. Nevertheless, THz irradiation may have an impact on the dimensional structure of proteins and nucleic acids of a higher order, resulting in changes to gene expression, certain metabolic pathway activities, and cell division systems [1].

All the above-mentioned concerns and the appearance of new emitting and detecting devices for THz irradiation have led to a dramatic increase in the number of studies in the last decades [2,3,4,5,6].

THz irradiation may impact both biological molecules directly and cell processes in general. The first type of impact is connected with the levels of macromolecule rotational and vibrational energy being overlaid with THz wave frequency characteristics. It has been found that a THz stimulus with a frequency of 44.0 THz accelerates the unwinding of DNA duplexes by at least 20-fold, and this effect is due to the breakage of weaker hydrogen bonds between purine bases as a result of the resonance between the THz electromagnetic waves and the vibrations of the chemical bonds of purines [7]. Moreover, resonant excitation can break the methyl–DNA bond, and this effect of decreased DNA methylation upon exposure to THz radiation (1.7 THz) has been demonstrated in vivo for various blood cancer cell lines and M-293T melanoma cells [8,9].

The emergence of information that double-stranded DNA breaks upon 10 min of exposure to pulsed THz radiation (0.1–2.0 THz) is evidenced by findings about a significant induction of histone H2AX phosphorylation in the cells of a human skin tissue model consisting of normal epidermal keratinocytes and dermal fibroblasts [10]. On the other hand, after the in vitro exposure of human embryonic fibroblasts to low-frequency THz radiation (0.1–0.15 THz), such effects as DNA damage and greater phosphorylation of histone H2AX were not observed, but there was an increase in the total number of micronuclei and centromere-positive micronuclei, indicating that THz radiation can lead to the loss of chromosomes [11]. The THz irradiation (2.3 THz) of human stem cells and the cells of the Enterobacteriaceae *Escherichia coli* and *Salmonella typhimurium* did not cause double-stranded breaks in chromosomes [12,13].

That is, the impact of THz radiation on the primary structure of DNA depends not only on the parameters of irradiation, but also on the cell type.

As for the effect of THz radiation on cellular processes, it also depends on many factors, including the duration and intensity of the irradiation as well as the experimental conditions and the origin of the cells. Evidence is accumulating about the effects of THz radiation on gene expression. For instance, the influence of pulsed radiation in the THz range (~0.8 THz) on induced pluripotent human stem cells manifests itself as changes in the expression of genes involved in the regulation of the actin cytoskeleton as well as gene networks that are under the control of Zn-dependent transcription factors, leading to cell cycle arrest in the G2/M phases and the disruption of the pathways of neuroepithelial cell differentiation [14].

Low-frequency THz radiation (0.46 THz) enhances the polymerization of purified actin in vitro, and by similarly affecting actin polymerization in vivo, inhibits human cell division. In an aqueous medium (~1 mm thick) at an irradiation frequency of 4 THz, both in vitro and in live cells, actin filaments disintegrated, possibly as the result of a shock wave [15,16,17]. The exposure of neuron-like PC 12 cells to THz radiation (0.3–19.5 THz) for 10 min temporarily increased the permeability of the cell membranes [18].

The prolonged pulsed broadband THz irradiation (within 10 THz) of mouse stem cells led to changes in the expression of genes encoding adiponectin, GLUT4, FABP4, and PPARG, with differentiation acceleration of these cells towards the fatty phenotype, which may have been due to the activation of transcription factor PPARG (peroxisome proliferator-activated receptor gamma) gene expression [19,20,21].

The THz irradiation of *E. coli* K12 JM109 cells for 15 min (1–2 THz) followed by a 10 min incubation activates the expression of genes responsible for the biosynthesis of pili and colanic acid, as well as genes that control cell division, thus giving rise to cells with an abnormal morphology that are prone to aggregation [1].

The use of biological sensors has also made it possible to reveal that the irradiation of *E. coli* cells with THz electromagnetic waves (0.14 THz) leads to the activation of gene networks controlling the stress response, assimilation of disaccharides, and metabolism of amino acids under the influence of the transcription factors YdeO, ChbR, and TdcR [22,23]. At a higher frequency (2.31 THz), however, the gene networks of oxidative stress are activated, and the homeostasis of transition metals is reinforced [24,25], as are biofilm formation processes controlled by transcription factor MatA [22].

A recent review (Sun et al., 2021) suggests that, while there has been some progress in the research on the effects of THz radiation on biological systems, there are still many technical obstacles, and one of them is accurate temperature control. It is believed that to detect nonthermal effects of THz radiation, the temperature difference from a control should not exceed 0.1 °C [5]. This is a major limitation for identifying the biological effects of THz radiation properly in temperature-sensitive organisms.

One possible way to solve this problem is to use organisms with a high resistance to temperature changes for studying the effects of THz radiation on living systems, e.g., thermophilic species of bacteria and archaea, which are adapted to a wide range of temperatures. This approach, however, does not obviate strict temperature control.

In this study, we investigated the effects of irradiation with electromagnetic waves in the THz range. To do so, we used cells of the thermophilic bacterium *Geobacillus icigianus*, which were isolated from silt samples at a steam-gas hydrothermal (97 °C) outlet located near the Troinoy geyser (Valley of Geysers, Kronotsky Nature Reserve, Kamchatka Peninsula, Russia) because it was shown that *G. icigianus* cells tolerate a wide temperature range well: from 50 °C to 75 °C, with an optimum of ~60 °C [26]. We found that in this bacterium, in response to 15 min of THz irradiation (2.3 THz), there are significant changes in the levels of proteins and enzymes involved in respiration, energy metabolism, the regulation of transcription and translation, and in various systems of cell defense against oxidative and other types of stress.

## 2. Results

### 2.1. Changes in the Proteomic Profile of the Thermophilic Bacterium G. icigianus in Response to Irradiation with THz Electromagnetic Waves (2.3 THz) for 15 Min

Table 1 and Table 2 show the results from a comparative analysis of electropherograms of the proteins isolated from the irradiated and control cultures of *G. icigianus*. Table 1 indicates that this type of THz irradiation led to a significant decrease in the concentration of 19 protein spots in the bacterial proteome, indicating the suppression of the processes governed by these proteins.

The identification of these proteins revealed, firstly, reduced levels of the α and β ETF subunits of flavoproteins (EP10_09770 and EP10_09765, which underlie the activity of the electron transport chain) and of NADH dehydrogenase (EP10_04625), which transfers two electrons from NADH to quinone in conjunction with the translocation of four protons across the cell membrane, i.e., this protein is capable of generating PMF (proton motive force). Under aerobic culture conditions, NADH dehydrogenase is the only enzyme with a proton translocation ability in this bacterium.

Secondly, there was a reduced level of a key enzyme in the glycolytic pathway of glucose utilization: glyceraldehyde-3-phosphate dehydrogenase (EP10_12960), which produces NADH and leads to the synthesis of pyruvate. It should be mentioned here that glyceraldehyde-3-phosphate dehydrogenase has multiple functions in the cell, and its downregulation under THz irradiation can affect not only the glycolytic pathway activity, but also the efficiency of such processes as DNA repair, translation elongation, and the degradation of RNA and proteins through interactions with various factors, as shown for the translation elongation factor EF-Tu and the molecular chaperone DnaK in *E. coli* and *B. subtilis* [27,28,29].

Thirdly, the activity of L-serine deaminase (EP10_16780) was lowered, which, during growth in a medium rich in amino acids and poor in sugars (as in our case), can be one of the main suppliers of carboxylic sources that ensure energy metabolism in the cell, by utilizing L-serine for the synthesis of pyruvate, as demonstrated in *E. coli* [30,31]. The availability of pyruvate plays a key role not only in the activation of the NADH-producing tricarboxylic acid (TCA) cycle, but also in the synthesis of a variety of amino acids and cofactors. D-3-phosphoglycerate dehydrogenase (EP10_12110), which produces NADH and implements de novo serine synthesis, turned out to be downregulated too.

To sum up, THz irradiation of *G. icigianus* cells for 15 min led to the partial inhibition of respiratory function and energy metabolism and possibly a decrease in the activity of enzymes that use NADH and pyruvate as substrates.

Fourthly, there was downregulation of the translation elongation factor Tu (which, in its GTP-bound state (EF-Tu), delivers aminoacyl tRNAs to the ribosome in the form of a ternary complex) as well as underexpression of the ribosome recycling factor (which ensures translation accuracy, the disassembly of the translational complex, and the reuse of ribosomes in subsequent cycles of translation [32]) and cysteine-tRNA ligase. Furthermore, there was a low concentration of transcription elongation factor GreA, which prevents the arrest of transcription during elongation and increases transcription accuracy [33]. It is thought that Gre proteins are also required for transcriptional homeostasis under thermal and mechanical stress [34], including the stress caused by THz irradiation [35].

These data indicate a decline in the efficiency and accuracy of transcription and translation in *G. icigianus* after THz irradiation for 15 min.

**Table 1:** The identified proteins found to be significantly downregulated in the proteomic profile of *G. icigianus* after 15 min of THz irradiation.

Protein/Reaction	Gene/Locus	Process/Function	∆
Electron transfer flavoprotein subunit beta/FixA family protein (ETFb) Electron transfer flavoprotein subunit alpha/FixB family protein (ETFa)	EP10_09770 EP10_09765	ETF:heterodimer, a key component of the electron transfer chain	1.7 1.4
NADH dehydrogenase (EC 7.1.1.2), NADH:ubiquinone reductase (H^+^-translocating) NADH + Q + 5H^+^-> QH_2_ + NAD +4H^+^	EP10_04625	Respiration, generates PMF	1.6
D-3-phosphoglycerate dehydrogenase (EC 1.1.1.95) G3P + NAD^+^ -> 3-phosphooxypyruvate + NADH + H^+^	EP10_12110	NADH synthesis, serine biosynthesis	1.8
Type I glyceraldehyde-3-phosphate dehydrogenase (NAD^+^), (EC 1.2.1.12) G3P + P_i_ + NAD -> NADH + 1, 3PDG	gap/ EP10_12960	Key enzyme of the glycolytic pathway, NADH synthesis	1.2
Class II fructose-bisphosphatase Fructose-1,6-bisphosphatase (FBPase) (EC 3.1.3.11) FDP+ H_2_O + ADP -> F6P + ATP	glpX/ EP10_10310	ATP biosynthesis, peptidoglycan biosynthesis	1.2
Elongation factor Tu	tuf/ EP10_11775	Translation elongation factor, GTPase	1.6
Ribosome recycling factor	frr/EP10_17125	Translation	1.4
Cysteine-tRNA ligase (EC:6.1.1.16)	CysS/ EP10_11880	Translation	1.35
Transcription elongation factor GreA	EP10_01545	Transcription, chaperone activity	1.8
Bifunctional 3,4-dihydroxy-2-butanone 4-phosphate synthase, DHBP synthase (EC 4.1.99.12)/GTP cyclohydrolase II (EC 3.5.4.25)	EP10_18260	Riboflavin biosynthesis, protects DNA from oxygen radicals	1.4
Thioredoxin reductase (EC 1.8.1.9) Thr + NADP^+^ -> Thr disulfide + NADPH + H^+^	EP10_04630	Antioxidant system, NADPH synthesis	1.6
Thiol peroxidase, Tpx-type (EC:1.11.1.24) [thioredoxin]-dithiol + a hydroperoxide -> [thioredoxin]-disulfide + alcohol + H_2_O	EP10_18085	Thiol peroxidase is part of an oxidative stress defense system	1.6
Deacetylase BshB2 (deacetylase of the LmbE family protein)	bshB2/ EP10_10565	Bacillithiol synthesis, resistance during oxidative stress	1.55
Pantoate-beta-alanine ligase (EC 6.3.2.1) ATP + pantoate + alanine -> AMP + diphosphate + pantothenate	EP10_07480	Synthesis of pantothenate from alanine	1.3
L-serine deaminase, L-serine ammonia-lyase, iron-sulfur-dependent, subunit beta (EC 4.3.1.17) L-serine -> pyruvate + NH_3_	sdaAB/ EP10_16780	Pyruvate synthesis	1.4
Acetyl-CoA acetyltransferase (EC 2.3.1.9) 2 Acetyl-CoA -> CoA + acetoacetyl-CoA	thlA/ EP10_03775	CoA biosynthesis	1.7
Acetate kinase (EC 2.7.2.1) ATP + acetate -> ADP + acetyl phosphate	ackA/EP10_18095	АсСоА biosynthesis	1.4
Pyridoxal 5′-phosphate synthase (PLP synthase) Glutaminase subunit PdxT (EC 2.4.2.-)	pdxT/ EP10_02655	De novo pyridoxal 5′-phosphate biosynthesis	1.5
Inorganic pyrophosphatase (EC 3.6.1.1) Diphosphate + H_2_O <-> 2 phosphate	EP10_12120	Cell growth	1.3

There was also downregulation of many proteins that contribute to the defense of the cell against oxidative stress, namely: deacetylase BshB2 (EP10_10565), which ensures the synthesis of bacillithiol (an analog of glutathione in Firmicutes [36,37]); thioredoxin reductase (EP10_04630), which effectively reduces the level of reactive oxygen species in the cell [38]; thiol peroxidase (EP10_18085), which is a part of the oxidative stress defense system [39]; and GTP cyclohydrolase II (EP10_18260, EC 3.5.4.25), which has an antimutator function and protects DNA from the adverse effects of endogenous oxygen radicals [40].

Two more enzymes should be mentioned whose downregulation under THz irradiation can overall negatively affect cell metabolism. This is DHBP synthase (EP10_18260, EC 4.1.99.12), which plays a key part in the biosynthesis of riboflavin (vitamin B2), a precursor of the cofactors FMN flavin mononucleotide and flavin adenine dinucleotide FAD [41], and pyridoxal 5′-phosphate synthase, performing the de novo synthesis of pyridoxal phosphate [42,43,44], which is a cofactor for ~4% of all enzymatic activities affecting the bacterial genome [45,46].

The possible impact of THz irradiation on the cell wall structure of *G. icigianus* is indicated by our finding of the downregulation of fructose-1,6-bisphosphatase (EP10_10315)—a key enzyme in *Bacillus* bacteria for the synthesis of fructose-6-phosphate, which is the main substrate for the synthesis of UDP-N-acetylmuramate (a peptidoglycan precursor) [47].

Finally, the level of inorganic pyrophosphatase (EP10_12120), which is an essential component of all living cells, was lowered. Blocking the expression of the inorganic pyrophosphatase gene leads to a halt in cell growth or the death of cells [48,49,50].

Thus, THz irradiation negatively affected the overall metabolism and growth of *G. icigianus* cells, e.g., through a decrease in translation efficiency and in the activity of the respiratory system, as well as in the synthesis of peptidoglycan and of cofactors NADH, FAD, and pyridoxal 5′-phosphate.

A list of the enzymes and proteins that were upregulated by the THz irradiation of *G. icigianus* cells for 15 min is given in Table 2.

Table 2 shows that this type of THz irradiation induced a significant increase in the concentration of 13 protein spots in the proteome of the bacterium *G. icigianus* after 15 min of irradiation, thus pointing to the activation of the processes performed by these proteins. These are, first of all, ATP synthase, which catalyzes the reversible reaction ADP + P_i_ + [H^+^]_ex_ ↔ ATP + H_2_O + [H^+^]_in_ and, during a PMF decline, works as an ATP-dependent proton pump. It is possible that under THz irradiation, when the level of proton-translocating NADH dehydrogenase diminished (Table 1), there was a compensatory overexpression of ATP synthase to maintain the PMF.

**Table 2:** The identified proteins whose levels significantly increased in the proteomic profile of *G. icigianus* after 15 min of THz irradiation.

Protein/Reaction	Gene/Locus	Process/Function	∆
ATP synthase alpha chain (EC 7.1.2.2) ADP + PI + [H^+^]_ex_ <-> ATP + H_2_O + [H^+^]_in_	atpA/ EP10_10185	ATP biosynthesis; when the PMF is decreased, ATP synthase catalyzes the reverse reaction, working as an ATP-dependent proton pump	1.3
Predicted L-lactate dehydrogenase (multisubunits, lldEFG, syn ykgEFG), PYR + NAD^+^ <-> LAC + NADH + H^+^	EP10_13275	NADH and lactate biosynthesis; iron–sulfur cluster-binding subunit YkgF [51]	1.6
Malic enzyme, NAD-dependent Mal + NAD -> Pyr + NADH + CO_2_	EP10_15870	NADH and pyruvate biosynthesis	1.8
Oxidoreductase, aldo/keto reductase family	EP10_11090	NADH and NADPH biosynthesis	1.55
2-Oxo acid dehydrogenase complex subunit E2 Dihydrolipoamide acyltransferase component of branched-chain alpha-keto acid dehydrogenase complex (EC 2.3.1.168)	EP10_06390	Complex catalyze-irreversible oxidation of 2-oxoacids with production of acyl-CoA and NADH	2.15
Aspartate-semialdehyde dehydrogenase (EC 1.2.1.11) L-aspartate 4-semialdehyde + phosphate + NADP+ -> L-4-aspartyl phosphate + NADPH + H^+^	Asd/ EP10_17245	NADPH biosynthesis	2.0
Isocitrate dehydrogenase [NADP] (EC 1.1.1.42) ICIT + NADP -> AKG + CO_2_ + NADPH	EP10_07280	NADPH biosynthesis	2.2
HslU—subunit of the ATP-dependent HslUV protease	EP10_16935	Molecular chaperone	1.3
Trehalose-6-phosphate hydrolase (EC 3.2.1.93) Trehalose 6-phosphate + H_2_O -> glucose + glucose 6-phosphate	EP10_08195/treA	Trehalose—osmoprotectant and carbon source [52]	2.0
Tyrosine-tRNA ligase (EC 6.1.1.1)	EP10_13885	Translation	1.9
Tripeptide aminopeptidase (EC 3.4.11.4)	pepT EP10_11320	Protease	1.6
Excinuclease ABC subunit UvrB, ATP-dependent	EP10_00715	DNA reparation	3.3
Cysteine methyltransferase Methylated-DNA-protein-cysteine methyltransferase (EC 2.1.1.63)	EP10_00085	Repair of methylated DNA	5.4
Flagellar motor switch phosphatase FliY	EP10_17020	Chemotaxis disorder	1.8

A larger amount of the HslU subunit of the ATP-dependent multimeric protease HslVU (EP10_16935), tripeptide aminopeptidase (EP10_11320) (components of systems with chaperone and protease activities), excinuclease ABC subunit UvrB (EP10_00715), and cysteine methyltransferase (EP10_00085) (enzymes involved in the repair of DNA, including methylated DNA) was shown. The activation of these systems indirectly indicates damage to proteins and DNA, as well as the demethylation of the latter during THz irradiation.

We noticed an increased concentration of enzymes of the aldo/keto reductase family (EP10_11090) using NAD^+^ and NADP^+^ as acceptors. Among the identified proteins, there were predicted L-lactate dehydrogenases (EP10_13275) and malic enzymes (EP10_15870), which produce NADH during the synthesis of lactate and pyruvate; their upregulation may be a compensatory response to the decrease in the activity of the glycolytic pathway and the low level of L-serine deaminase, which produces pyruvate (Table 1).

An increase in the level of trehalose-6-phosphate hydrolase (EP10_08195) indicates the activation of trehalose utilization under THz irradiation, but it is difficult to explain this phenomenon because trehalose has two functions. If endogenously synthesized trehalose is used in this process, then its protective role under various types of stress decreases [53,54,55]; if trehalose coming from outside through the PTS system is used in the above process, then this results in a higher level of glucose in the cell and may be a response of the cell to the lowered activity of the glycolytic pathway and to the low level of L-serine deaminase, which produces pyruvate (Table 1).

Lastly, the THz irradiation for 15 min upregulated NADP-dependent isocitrate dehydrogenase (EP10_07280) and aspartate-semialdehyde dehydrogenase (EP10_17245), which increase the availability of NADPH. This result may be due to various aspects of the influence of THz radiation on cell metabolism. Firstly, the finding may be due to the cell’s response to a possible decline in the amount of riboflavin, as mentioned above; the synthesis of riboflavin depends on NADPH availability [56]. The second possible explanation is the cell’s response to a probable decrease in the synthesis of peptidoglycan, which underlies the structure of the bacterial cell wall; this is because the initial stages of the synthesis of the bacterial cell wall require NADPH availability [57]. This diminution, as presented in Table 1, may be related to a lower concentration of fructose-bisphosphatase (class II, *glpX*, EP10_10315), whose activity determines the synthesis of fructose-6-phosphate, which in *Bacillus* bacteria is the main substrate for the synthesis of UDP-*N*-acetylmuramate, a peptidoglycan precursor [47].

It is also worth mentioning the reaction of flagellar motor switch phosphatase FliY (EP10_17020) to THz irradiation; this protein is located within the complex of flagellar switches, and the rotational function of the flagella depends on its activity [47]. The observed upregulation of FliY points to an impairment of this function of flagella and, consequently, an impairment of the chemotaxis of *G. icigianus* under THz irradiation.

### 2.2. The Reaction of G. icigianus Cells 10 Min after the End of the THz Irradiation (2.3 THz)

Data obtained from the comparative analysis of electropherograms of the proteins isolated from the irradiated and control cultures of *G. icigianus* 10 min after the end of the irradiation are presented in Table 3 and Table 4. The comparison of these data and those presented above revealed that 10 min after the end of THz irradiation, most of the proteins and enzymes present in Table 1 and Table 2 were now absent in Table 3 and Table 4. This means that their concentrations did not differ from those in the control culture of *G. icigianus*. For instance, after 10 min, the levels of the components of systems with chaperone, protease, nuclease, and repair activities (DnaK, HslU, UvrB, PepT, and cysteine methyltransferase) decreased to the control level (and below) (compare Table 2 and Table 3), indirectly indicating the rapid restoration of the structure of proteins and DNA after the end of the THz irradiation. At the same time, there was a recovery of the systems that protect the cell from oxidative stress, with the exception of GTP cyclohydrolase II/DHBP synthase (Table 1 and Table 3), which has a dual function and, in addition to protecting DNA from the effects of oxygen radicals [40], takes part in riboflavin synthesis [41].

The proton translocation function, controlled by NADH dehydrogenase, also proved to be restored. The slight decrease in the amount of ATP synthase (Table 4) 10 min after the irradiation may be attributed to the restoration of the NADH dehydrogenase level, and consequently, the PMF.

The level of inorganic pyrophosphatase returned to baseline (Table 1) (the lack of its activity suppressed cell growth [48,49]), as did the level of flagellar motor switch phosphatase FliY (Table 2), whose increased activity can switch off chemotaxis and the rotational function of flagella [58]. The translation and transcription efficiency and accuracy—controlled by the ribosome recycling factor and the transcription elongation factor GreA—partially recovered too.

The exceptions were the flavoproteins (ETFa/b) of the electron transport chain, the translation elongation factor Tu, the bifunctional enzyme DHBP synthase/GTP cyclohydrolase II (which, in addition to its antimutator function, plays a key part in riboflavin biosynthesis), and acetyl-CoA acetyltransferase (using acetyl-CoA for CoA biosynthesis). Their levels in the cell remained low even 10 min after the end of the THz irradiation (Table 1 and Table 3).

Furthermore (Table 3), there was underexpression of RpoA polymerase (EP10_11630, a key enzyme that initiates transcription) and of methionine adenosyltransferase (EP10_09910, which converts L-methionine into S-adenosyl-L-methionine: a universal donor of methyl groups) [59]. Phosphopentomutase (EP10_18365) was found to be downregulated as well; it performs a catabolic function in relation to deoxyribonucleosides, and in *Bacillus* bacteria, is one of the three main enzymes for the utilization of nucleosides and their use as an energy source [60]. Additionally, there was downregulation of proteins that implement the import of amino acids (EP10_06290), which, during the growth in the LB medium, are also used by the cell as energy sources. There was a reduced level of acid sugar phosphatase (EP10_04705) (a TIGR01457 family HAD-type hydrolase), which releases inorganic phosphate from sugar metabolites containing a phosphate group.

This means that the processes controlled by these proteins, including translation and transcription, respiration, and energy metabolism, are sensitive to THz irradiation and have not had enough time to fully recover 10 min after the irradiation cessation.

At the same time, as evident in Table 4, 10 min after the end of THz irradiation, the upregulation of a rather wide range of enzymes producing NADH and NADPH was observed in the *G. icigianus* cell, but this list differs from that seen in Table 2. For example, there were elevated levels of NADH-producing glyceraldehyde-3-phosphate dehydrogenase (EP10_12960), pyruvate dehydrogenase (EP10_14290), leucine dehydrogenase (EP10_06415), glycine dehydrogenase (EP10_05755), and glutamate dehydrogenase (EP10_12170). With the exception of glyceraldehyde-3-phosphate and pyruvate dehydrogenase, the other mentioned enzymes participate in the metabolism of amino acids, whose utilization during the growth in the LB medium also led to the production of carbon and nitrogen sources for the cell and contributed to the maintenance of general metabolism [61]. Apparently, this can explain the increased level of arginase too (EP10_17840), which produces ornithine and urea, the subsequent utilization of which also results in the production of carbon and nitrogen sources [62].

In contrast, the upregulation of enzymes utilizing the amino acids leucine, glycine, glutamate, and arginine may be a protective reaction of the cell to THz irradiation because this reaction raises the intracellular ammonium content (Table 4) and promotes the neutralization of protons arising from the irradiation.

As for pyruvate dehydrogenase and glyceraldehyde-3-phosphate dehydrogenase, their upregulation 10 min after the irradiation indicates a cell response aimed at restoring energy metabolism after THz irradiation through a higher availability of pyruvate and the activation of the TCA cycle via acetyl-CoA formation. This supposition is supported by our data on the upregulation of phosphoenolpyruvate carboxykinase (EP10_09915, whose activity enlarges the pyruvate pool; Table 4) and the still-low level of acetyl-CoA acetyltransferase (EP10_03775), which uses acetyl-CoA for other purposes (Table 1 and Table 3).

We think that the maintenance of a high expression of numerous enzymes that produce NADH and the observed tendency for activation of the TCA cycle (through an increase in the synthesis of acetyl-CoA and phosphoenolpyruvate) are the cell’s responses to the suppression of the energy and respiratory systems during the THz irradiation and to their incomplete recovery 10 min after termination of the irradiation. This notion is corroborated by the impairment of the amino acid transport system because of the observed downregulation of relevant transporter proteins (EP10_06290) (Table 3), the activity of which is important for maintaining metabolism during bacterial growth in the LB medium.

As for the production of NADPH, at the previous data point, it was enhanced by two NADP-dependent enzymes: aspartate-semialdehyde dehydrogenase (EP10_17245) and isocitrate dehydrogenase (EP10_07280) (Table 2). At 10 min after the irradiation, the level of aspartate-semialdehyde dehydrogenase was still elevated; besides, the amount of 6-phosphogluconate dehydrogenase (EP10_18520), which produces NADPH, went up.

Here, we would like to draw the reader’s attention to 6-phosphogluconate dehydrogenase, which catalyzes the synthesis of ribulose-5-phosphate and NADPH from 6-phosphogluconate and NADP. Both metabolites are substrates for the biosynthesis of riboflavin, and it is known that the activation of glucose utilization via the pentose phosphate pathway (whose component is 6-phosphogluconate dehydrogenase) enhances the synthesis of riboflavin [56]. Nonetheless, we cannot say that the amount of riboflavin in the *G. icigianus* cells recovered 10 min after THz irradiation, because the level of DHBP synthase was still low (Table 3).

## 3. Discussion

The analysis of the data presented in Table 1, Table 2, Table 3 and Table 4 shows that the specific feature of the thermophilic bacterium *G. icigianus*’s response to THz irradiation was impaired activities of the electron transport chain, cellular metabolism, and some components of the translation system. This observation was confirmed by the rapid and sustained activation of the cellular systems that compensate for the inhibition of the respiratory and glycolytic systems, namely the upregulation of ATP synthase and of a wide range of enzymes that generate NADH. With regards to the translation system, a similar effect was previously shown when cells of the thermostable archaea *Halorubrum saccharovorum* were exposed to long-term, non-thermal THz radiation (2.3 THz, 5 h), where the expression of genes encoding the structure of translation initiation and elongation factors was also reduced by irradiation [63].

The overall suppression of cellular metabolism and growth was evidenced by the downregulation of the enzymes that control the synthesis of the cofactors NADPH, FAD, and pyridoxal 5′-phosphate and of the enzymes participating in the synthesis of riboflavin, oxaloacetate, and peptidoglycan. Another piece of supporting evidence is the cell’s response aimed at restoring metabolism and growth: e.g., the partial activation of the TCA cycle and the partial upregulation of enzymes that produce NADPH.

We have suggested that the upregulation of enzymes that raise the availability of NADPH may reflect the problems associated with the inhibition of *G. icigianus* growth during THz irradiation, including the consequences of diminished riboflavin synthesis (Table 1 and Table 3) as mentioned above, and the possible impairments of peptidoglycan synthesis, which depends on NADPH availability (whose initial synthesis steps also require NADPH) [57], and the cell wall structure. The possible disturbances of the processes involved in cell wall formation were evidenced by several findings. Firstly, during irradiation, the level of L-serine deaminase (EP10_16780, which utilizes serine) decreased (Table 1), and secondly, after the irradiation termination, the level of serine hydroxymethyltransferase (EP10_10230, which synthesizes serine) significantly increased (Table 4). A potential consequence of these alterations is a higher intracellular concentration of serine and a greater probability of its incorporation into peptidoglycan instead of alanine; this substitution is expected to disturb the peptidoglycan synthesis and weaken the cell wall [64].

Furthermore, it was shown that ATPase HslU, being a molecular chaperon, prevents cell growth inhibitor SulA aggregation and its elimination via HslV-mediated degradation [59,63]. Cell growth deceleration may be a consequence of the increased concentration of HslU during THz irradiation.

Meanwhile, the restoration of the levels of inorganic pyrophosphatase and fructose-1,6-bisphosphatase (necessary for the synthesis of the peptidoglycan precursor UDP-N-acetylmuramate [58]) and the overexpression of other enzymes of peptidoglycan synthesis, UDP-*N*-acetylmuramoyl-tripeptide-D-alanyl-D-alanine ligase (EP10_18840) in particular, point to the partial recovery of the processes involved in cell growth and peptidoglycan synthesis 10 min after the termination of irradiation. On the whole, these results suggest that the processes associated with cell wall synthesis and *G. icigianus* growth are sensitive to THz irradiation.

On the other hand, *G. icigianus* cells proved to be sufficiently resistant to stress and demonstrated a rapid recovery (to baseline) of the systems with chaperone, protease, nuclease, and antioxidant activities, as well as the partial restoration of the accuracy and efficiency of the translation system 10 min after the end of THz irradiation, in contrast to mesophilic *E. coli*, whose antistress gene systems remain active even 10 min after THz irradiation [23,24].

It should also be noted that in *G. icigianus* cells, there is the possibility of DNA demethylation after THz irradiation, as has been shown on eukaryotic cells [8,9]. In *G. icigianus*, this possibility may be due to the activation of two enzymes, excinuclease ABC subunit UvrB, which is ATP-dependent (EP10_00715), and methylated-DNA-protein-cysteine methyltransferase (EP10_00085) (Table 2), which is involved in the repair of DNA and its methylated fragments. Indeed, DNA methylation in bacteria is commonly associated with restriction-modification systems that act as key moderators of horizontal gene transfer [65]. DNA methylation plays an important role in bacterial biology: phenomena such as the DNA replication time, chromosome division into daughter cells, DNA repair, transposition time, and conjugal plasmid transfer are sensitive to the methylation states of specific DNA sites [66,67,68]. The activation of cysteine methyltransferase (EC 2.1.1.63) confirms the epigenetic switching of gene expression after THz exposure.

## 4. Materials and Methods

The experiments were conducted under the conditions of strict temperature control in experimental and control samples; therefore, the observed changes in the proteomic profile were caused by the nonthermal effect of THz radiation. Heat shock is known to induce the expression of several heat shock proteins. The nonthermal nature of the THz irradiation was corroborated by the absence of heat shock proteins among the protein spots found to be upregulated in the experimental group.

### 4.1. Cell Culture Conditions

A pure culture of the Gram-positive thermophilic bacterium *G. icigianus*, strain G1w1, was used from the collection at the ICG SB RAS, where the microbe was stored at 4 °C. To switch the bacterium to its active state, the Luria–Bertani (LB) culture medium (NaCl 5 g, yeast extract 5 g, tryptone 10 g in water per 1000 mL of medium) with and without agar was employed. The suspensions of growing cells were sub-cultured into Petri dishes and 50 mL tubes. Cultivation in the Petri dishes (i.e., culture plates) was carried out in a Memmert INB 200 laboratory thermostat at 60 °C. Cultivation in a liquid medium was performed at 60 °C and 250 rpm in an orbital shaker-incubator (Biosan ES20/60).

For each experiment, a *G. icigianus* strain culture recovery from the microorganism collection of IC&G SB RAS was performed. To obtain single colonies, a bacterial culture was seeded on the agar LB medium. A single colony was placed in 5 mL of the liquid LB medium in a 15 mL tube to prepare an overnight culture. The latter was transferred to a 250 mL flask with 100 mL of the LB medium for further incubation.

An Epoch spectrophotometer (Biotek) was used to determine the optical density of the bacterial culture. The measurement was performed in a 96-well plate.

According to the results of the measurements, a bacterial culture that was grown from a single colony in the LB medium and that reached an optical density of 0.5 was frozen in aliquots of 400 μL in 10% glycerol for storage at –70 °C. For subsequent use in the experiment, 200 μL from one aliquot of the frozen culture was added to 5 mL of the LB medium. After 18 h of cultivation, the culture was transferred into a flask with 100 mL of the LB medium and incubated until an optical density of 0.5 was reached. To obtain the material for one biological replicate, three culture flasks with the bacterial cell suspension were set up with an interval of 3 h.

### 4.2. Irradiation Conditions

The experiments were conducted at the Novosibirsk Free Electron Laser (NovoFEL) facility of the Siberian Synchrotron and Terahertz Radiation Centre, which has been designed and put into operation by Budker Institute of Nuclear Physics SB RAS (Novosibirsk, Russia). The NovoFEL irradiation settings were as follows:

Wavelength, mm        0.05–0.340
Pulse duration, ps         50
Pulse repetition frequency, MHz 2.8–11.2
Average power, W        up to 400
Peak power, MW        up to 1
Minimum relative line width   3 × 10^−3^

For the experiment, 60 μL of the bacterial culture in the LB medium was transferred into a specially designed cuvette, which has been described previously [25]. The cells were exposed to THz radiation with a power density of 0.23 W/cm^2^ and a wavelength of 130 μm, while the temperature of the medium in the cuvette was maintained at 60 °C ± 1 °C and monitored using a TKVr-SVIT101 high-precision thermal imager (Institute of Semiconductor Physics SB RAS, Novosibirsk, Russia) with an accuracy of 0.03 °C. The irradiation duration was 15 min. Then, the culture was transferred into a microcentrifuge tube and either frozen in liquid nitrogen to assess the rapid proteomic response or incubated in a thermostat at 60 °C and 250 rpm for 10 min to let the response develop, after which the cells were frozen in liquid nitrogen. In parallel, as a control, 60 μL of a bacterial culture in the LB medium was transferred into a specially designed cuvette (identical to the one above), placed in a thermostat at 60 °C, and incubated for 15 min. Next, the culture was either transferred into a microfuge tube and frozen in liquid nitrogen to characterize the rapid proteomic response or incubated in a thermostat at 60 °C and 250 rpm for 10 min to let the response develop, after which the cells were frozen in liquid nitrogen. For one biological replicate, the material from the three flasks was combined after one day of irradiation (see the end of Section 2.1). The material was collected from each flask after 3 h (with even alternation of the samples) to investigate the rapid proteomic response to THz radiation and to study the further development of the response to THz radiation during a subsequent 10 min incubation of the culture.

### 4.3. Two-Dimensional Electrophoresis of Proteins

The adaptation of *G. icigianus* to extreme environmental conditions makes its proteins difficult to isolate and separate during a proteomic analysis [69,70,71]. For the two-dimensional electrophoresis of proteins from thermophilic bacteria, a technique was developed by taking into account the difficulties with the separation of such bacterial proteins.

To isolate the total protein, *G. icigianus* cells were pelleted by centrifugation at 2300× *g* for 3 min at 4 °C. The pellet was washed twice with a NaCl solution of the same concentration as in the medium. After that, 200 μL of lysis buffer of the following composition was added to the pellet: 7 M urea, 2 M thiourea, 4% CHAPS, 65 mM DTT, 0.4% ampholyte pH 3–10 (BioRad, Hercules, CA, USA), and 1 mM phenylmethylsulfonyl fluoride.

The sample was ultrasonicated in a pulsed mode (2 s ON/10 s OFF) using a submerged ultrasonic processor probe (Antylia Scientific, Vernon Hills, IL, USA). The total ultrasonication time was 98 s (10 pulses) at an ultrasound frequency of 20,000 Hz while the test tube with the bacterial lysate was kept on ice. For the final protein extraction, the samples were incubated on ice for 15 min, followed by centrifugation at 16,100× *g* for 15 min at 4 °C. The supernatant was subjected to the proteomic analysis. The total protein concentration in the samples was determined by the Bradford method [72]. For the application of each sample to an isoelectrofocusing strip, 70 μg of the total protein was used. Isoelectric focusing was performed on 17 cm strips (pH 4–7) in a Protean IEF Cell (BioRad) under the following conditions: overnight passive rehydration, then 500–500 V, 1 h; 500–2500 V, 1 h; 3500 V, 3 h; and 3500–52,000 V, 1 h at 16 °C.

After reduction with 130 mM DTT and alkylation with 200 mM iodoacetamide, the proteins were separated in the second direction in SDS 12% polyacrylamide gels (acrylamide/bisacrylamide ratio 37.5:1) at a current of 25 mA per gel in a PROTEAN II xi Multi-Cell Electrophoresis Chamber (BioRad, Hercules, CA, USA).

The gels were stained with the Sypro Ruby fluorescent dye, giving a linearly proportional intensity of protein staining relative to the protein concentration in the gel, thereby enabling researchers to evaluate the relative intensity of the expression and visualize it by means of a VersaDoc MP4000Gel Documentation System (Bio-Rad, Hercules, CA, USA). A comparison of the obtained electropherograms was carried out using the PDQuest Advanced 2-D Analysis Software (Bio-Rad, Hercules, CA, USA).

**Figure 1:**
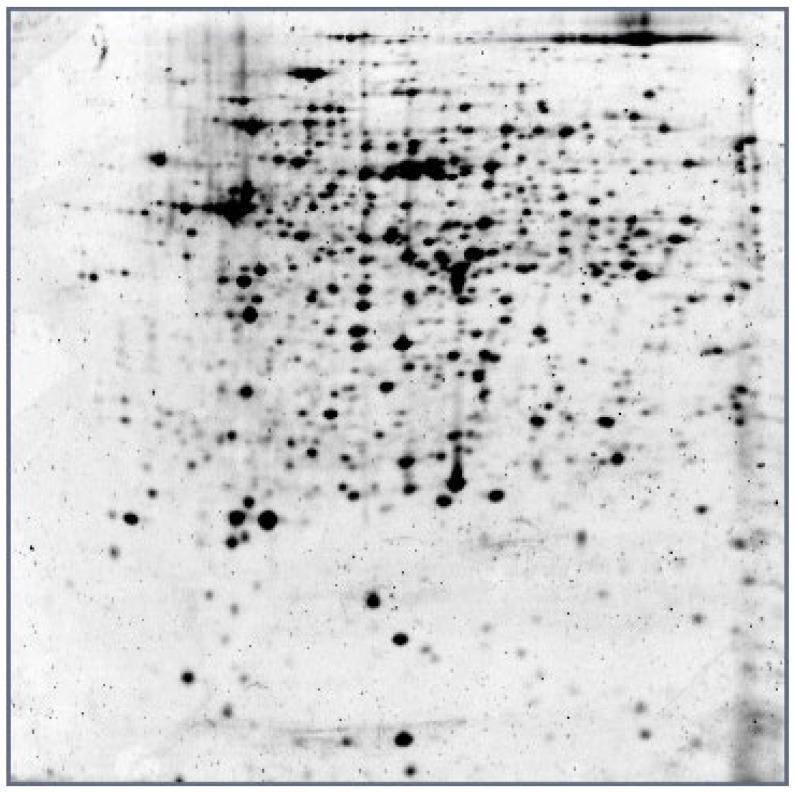
An electropherogram of *Geobacillus icigianus* G1w1 proteins. The strips (first direction) were 17 cm long (pH of 4–7); the SDS 12% polyacrylamide gel (second direction) was subjected to Sypro Ruby staining.

An electropherogram of *G. icigianus* proteins is shown in Figure 1.

### 4.4. Determination of the Proteomic Profile

Protein spots whose concentration significantly changed by a factor of 1.2 or more (*p* ≤ 0.05 in Student’s *t* test with a PLS (partial least squares) regression result of 98%) were excised from the gel by means of an EXQuest Spot Cutter (Bio-Rad, Hercules, CA, USA), then washed with 0.2 M ammonium bicarbonate in 50% acetonitrile at 37 °C for 20 min and subjected to tryptic digestion at 37 °C for 16 h with modified porcine trypsin (Trypsin Gold, Mass Spectrometry Grade, Promega, Madison, WI, USA) at a concentration of 0.02 mM in a buffer consisting of 40 mM bicarbonate ammonium in 10% acetonitrile (Merck, Rahway, NJ, USA). For the extraction of the resultant peptides, 20 μL of 5% trifluoroacetic acid (Sigma, St. Louis, MO, USA) was added into each microfuge tube and incubated for 45 min on a thermal shaker (Biosan, Warren, MI, USA) at 37 °C. Isolation and purification of the tryptic peptides were performed with a Millipore ZIPTIP C18 (Millipore, Burlington, MA, USA). Mass-spectrometric identification of the obtained peptides was performed on an Ultraflex III Tof/Tof instrument (Bruker Daltonics, Billerica, MA, USA) via matrix-assisted laser desorption ionization. The purified peptides were mixed with a saturated solution of a matrix: α-cyano-4-hydroxycinnamic acid (Bruker, Bremen, Germany) in 70% acetonitrile (Merck) supplemented with 0.1% trifluoroacetic acid (Sigma). An aliquot (1 μL) of the resulting mixture was applied to the mass spectrometer’s source and air dried. The analyses were carried out with the following instrument settings: accelerating voltage 25 kV, reflectron voltage 26.3 kV, mass range 500–3500 Da, and extraction delay 200 ns. Calibration was performed using a standard tryptic digest of bovine serum albumin (Bruker, Bremen, Germany) having the following peptide masses: 937.48, 1163.63, 1283.71, 1305.71, 1399.69, 1439.81, 1479.79, 1567.74, 1639.93, 1724.84, 1880.92, 1907.92, and 2045.02 Da. Proteins were identified by the Mascot search algorithm (http://matrixscience.com/home.html, accessed on 26 June 2022) using peptide mass fingerprint data. The search parameters were set as follows: max mass error, ±0.05 Da; the number of cleavage sites missed by trypsin, 1; possible modifications, methionine oxidation and cysteine carbamidomethylation; database, SwissProt.

To correctly identify proteins that change the expression level under the nonthermal action of THz radiation and to determine their functional roles in the *G. icigianus* genome, we utilized the results of our previously published genome-wide sequencing [73,74].

Interventionary studies involving animals or humans, and other studies that require ethical approval, must list the authority that provided approval and the corresponding ethical approval code.

## 5. Conclusions

Research on the effects of THz radiation on living organisms is currently underrepresented. In the present work, we showed that non-thermal THz radiation affects various metabolic pathways in the extremophilic *Geobacillus icigianus*, including disruptions to cell wall synthesis, the central metabolism, transcription, translation, and electron transport. In the short-term response, levels of the metabolic systems for electron transport; the regulation of transcription and translation; cell growth and chemotaxis; the synthesis of peptidoglycan, riboflavin, NADH, FAD, and pyridoxal phosphate cofactors; the Krebs cycle; ATP synthesis; chaperone and protease activity; and DNA repair systems were affected. For the long-term response, the effect of THz radiation was shown to be reversed in the systems of the cell’s anti-stress defense, chemotaxis and, partially, cell growth, but not for respiration and energy metabolism, the biosynthesis of riboflavin, cofactors, peptidoglycan, translation, and amino acid metabolism. The authors imply that the application of thermophilic bacteria as THz radiation objects allows the avoidance of the restrictions of THz radiation’s thermal effects.

## Figures and Tables

**Table 3:** The identified proteins significantly downregulated in the proteomic profile of *G. icigianusat* 10 min after the end of THz irradiation.

Protein/Reaction	Gene/Locus	Process/Function	∆
Electron transfer flavoprotein subunit alpha/FixB family protein ETFb Electron transfer flavoprotein subunit beta/FixA family protein, ETFa	EP10_09770 EP10_09765	ETF—heterodimer, a key component of the electron transfer chain	1.7 1.3
Elongation factor Tu	tuf/EP10_11775	Translation elongation factor, GTPase	1.4
DNA-directed RNA polymerase subunit alpha	rpoA/EP10_11630	Transcription	2.2 *
NAD-dependent malic enzyme Mal + NAD -> Pyr + NADH + CO_2_	EP10_15870	NADH and pyruvate biosynthesis	1.3 *
Acetyl-CoA acetyltransferase (EC 2.3.1.9) 2 Acetyl-CoA -> CoA + acetoacetyl-CoA	thlA/EP10_03775	Acetyl-CoA degradation	1.5 *
Molecular chaperone DnaK	EP10_05360	Molecular chaperone	1.35 *
Phosphopentomutase (EC 5.4.2.7) (2-Deoxy)-alpha-D-ribose 1-phosphate -> (2-deoxy)-D-ribose 5-phosphate	EP10_18365	Ribonucleoside catabolism	1.6
Methionine adenosyltransferase (EC 2.5.1.6) ATP + L-methionine + H_2_O -> phosphate + diphosphate + S-adenosyl-L-methionine	metK/EP10_09910	S-Adenosyl-L-methionine biosynthesis, SAM—methyl donor	1.4
Acid sugar phosphatase (TIGR01457 family HAD-type hydrolase)	EP10_04705		1.5
Amino acid ABC transporter ATP-binding protein	EP10_06290	Amino acid transport	1.5
Bifunctional 3,4-dihydroxy-2-butanone 4-phosphate (DHBP) synthase (EC 4.1.99.12)/GTP cyclohydrolase II (EC 3.5.4.25)	EP10_18260	Riboflavin biosynthesis, protects DNA from oxygen radicals	2.1 *

* The comparison involves two data points.

**Table 4:** The identified proteins significantly upregulated in the proteomic profile of *G. icigianus* at 10 min after the end of THz irradiation.

Protein/Reaction	Gene/Locus	Process/Function	∆
ATP synthase alpha chain (EC 7.1.2.2) ADP + PI + [H^+^]_ex_ <-> ATΦ + H_2_O + [H^+^]_in_	atpA/EP10_10185	ATP biosynthesis	1.1 ± 0.04
Type I glyceraldehyde-3-phosphate dehydrogenase (NAD^+^), EC 1.2.1.12 G3P + PI + NAD -> NADH + 1, 3PDG	gapA/ EP10_12960	NADH synthesis	1.3
Pyruvate dehydrogenase (Acetyl-transferring) E1 component subunit alpha (EC 1.2.4.1) PYR + NAD + COA -> ACCOA + NADH + CO_2_	pdhA/EP10_14290	Complex production of NADH and AcCoA	2.5
Dihydrolipoamide acetyltransferase component of pyruvate dehydrogenase complex, subunit E2 (EC 2.3.1.12) ACLIPO + COA -> ACCOA + DLIPO	EP10_14300	Complex production of NADH and AcCoA	2.8
2-Oxo acid dehydrogenase complex subunit E2 Dihydrolipoamide acyltransferase component of branched-chain alpha-keto acid dehydrogenase complex (EC 2.3.1.168)	EP10_06390	Complex production of acyl-CoA and NADH	1.6
Leucine dehydrogenase, NAD (+)-dependent (EC 1.4.1.9) L-leucine + H_2_O + NAD^+^ -> 4-methyl-2-oxopentanoate + NH_3_ + NADH + H^+^	EP10_06415	NADH biosynthesis	1.3
Glutamate dehydrogenase, NAD-specific (EC 1.4.1.2) L-glutamate + H_2_O + NAD^+^ -> 2-oxoglutarate + NH_3_ + NADH + H^+^.	EP10_12170	NADH biosynthesis	1.3
Glycine dehydrogenase (decarboxylating) (glycine cleavage system P1 protein) (EC 1.4.4.2) Gly + THF + NAD^+^ -> MetTHF + CO_2_ + NH_3_ + NADH	EP10_05755	NADH biosynthesis	1.6
Aspartate-semialdehyde dehydrogenase (EC 1.2.1.11) L-aspartate 4-semialdehyde + phosphate + NADP+ -> L-4-aspartyl phosphate + NADPH + H^+^	Asd/EP10_17245	NADPH biosynthesis	1.2
6-Phosphogluconate dehydrogenase, NADP (+)-dependent (EC 1.1.1.44) 6PG + NADP -> D-ribulose 5-phosphate + CO_2_ + NADPH	EP10_18520	PPP, NADPH biosynthesis	2.2
Phosphoenolpyruvate carboxykinase (ATP) (EC 4.1.1.49) ATP + oxaloacetate -> ADP + phosphoenolpyruvate + CO_2_	pckA/EP10_09915	Enhances pyruvate pool	1.3
Serine hydroxymethyltransferase (EC 2.1.2.1) MetTHF + glycine + H_2_O -> THF + L-serine	EP10_10230	L-serine biosynthesis	1.8
UDP-*N*-acetylmuramoyl(AM)-tripeptide-D-alanyl-D-alanine ligase, ATP-dependent (EC 6.3.2.10) ATΦ + UDP-*N*-AM-tripeptide + D-alanyl-D-alanine -> ADP + Pi + *N*-AM-pentapeptide	MurF/EP10_18840	Peptidoglycan biosynthesis	1.4
Arginase (L-arginine amidinohydrolase, EC 3.5.3.1) L-arginine + H_2_O -> L-ornithine + urea	rocF/EP10_17840	Urea and ornithine can be further utilized as nitrogen and carbon sources	1.3
Aspartate-tRNA ligase, Aspartyl-tRNA synthetase (EC 6.1.1.12)	EP10_01675	Translation	1.3
Proline-tRNA ligase, Prolyl-tRNA synthetase (EC 6.1.1.15)	EP10_17150	Translation	1.7

## Data Availability

The authors confirm that the data supporting the findings of this study are available within the article.
